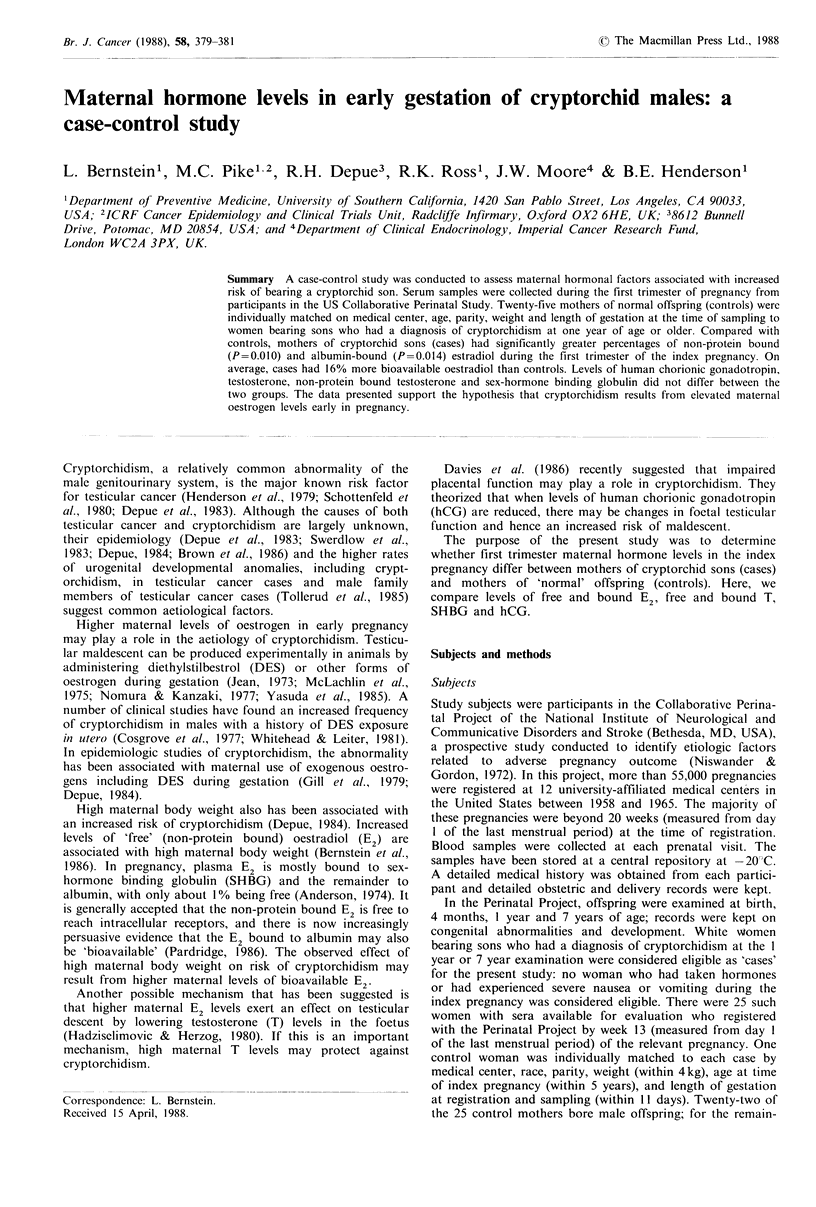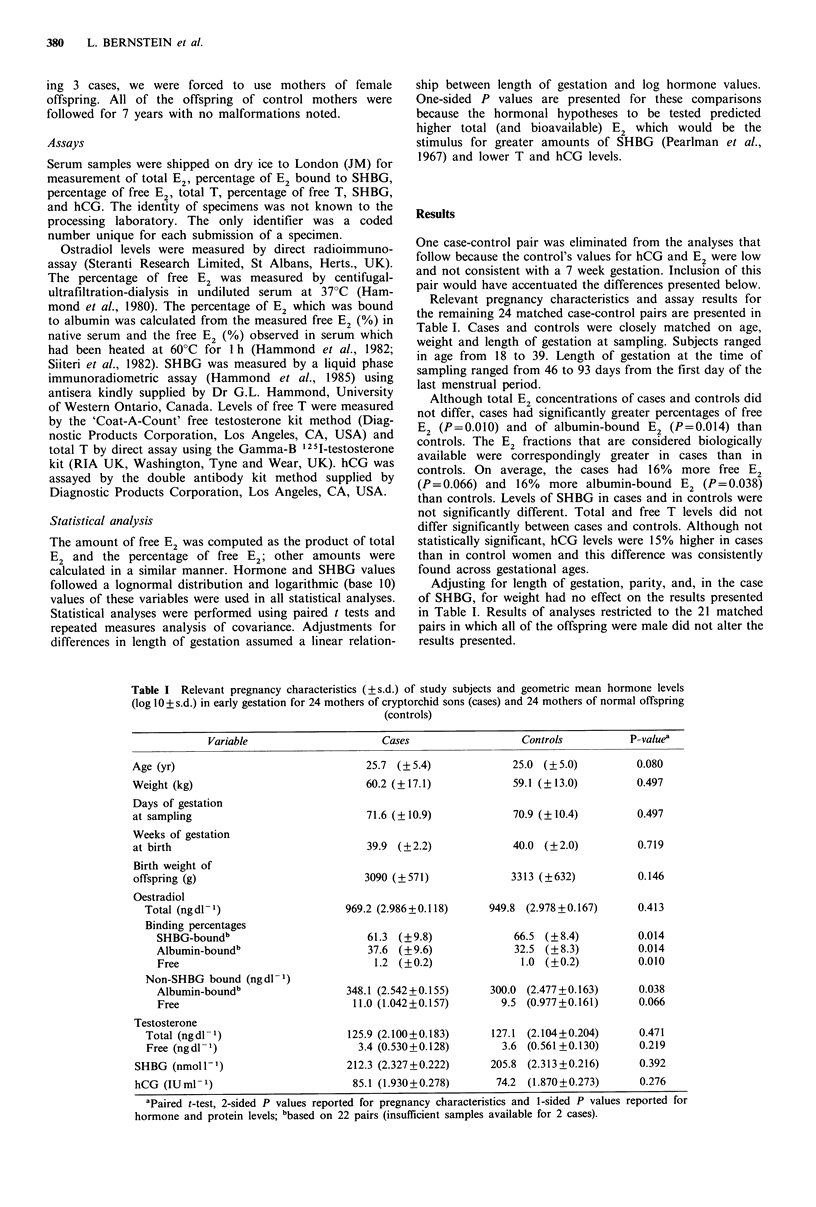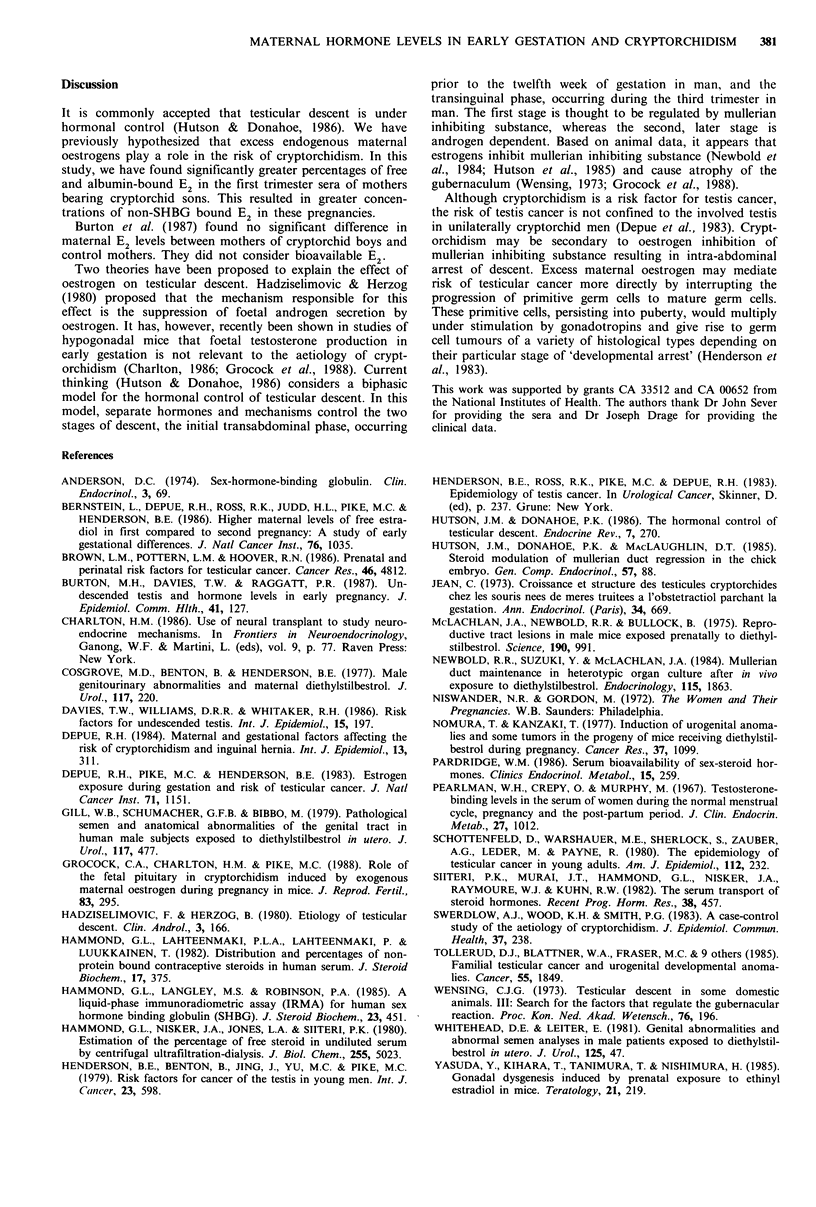# Maternal hormone levels in early gestation of cryptorchid males: a case-control study.

**DOI:** 10.1038/bjc.1988.223

**Published:** 1988-09

**Authors:** L. Bernstein, M. C. Pike, R. H. Depue, R. K. Ross, J. W. Moore, B. E. Henderson

**Affiliations:** Department of Preventive Medicine, University of Southern California, Los Angeles 90033.

## Abstract

A case-control study was conducted to assess maternal hormonal factors associated with increased risk of bearing a cryptorchid son. Serum samples were collected during the first trimester of pregnancy from participants in the US Collaborative Perinatal Study. Twenty-five mothers of normal offspring (controls) were individually matched on medical center, age, parity, weight and length of gestation at the time of sampling to women bearing sons who had a diagnosis of cryptorchidism at one year of age or older. Compared with controls, mothers of cryptorchid sons (cases) had significantly greater percentages of non-protein bound (P = 0.010) and albumin-bound (P = 0.014) estradiol during the first trimester of the index pregnancy. On average, cases had 16% more bioavailable oestradiol than controls. Levels of human chorionic gonadotropin, testosterone, non-protein bound testosterone and sex-hormone binding globulin did not differ between the two groups. The data presented support the hypothesis that cryptorchidism results from elevated maternal oestrogen levels early in pregnancy.


Br. J. Cancer (1988), 58, 379–381 © The Macmillan Press Ltd., 1988

### Maternal hormone levels in early gestation of cryptorchid males: a case-control study

L. Bernstein[1], M.C. Pike[1,2], R.H. Depue[3], R.K. Ross[1], J.W. Moore[4] & B.E. Henderson[1]

[1]*Department of Preventive Medicine, University of Southern California, 1420 San Pablo Street, Los Angeles, CA 90033, USA;* [2]*ICRF Cancer Epidemiology and Clinical Trials Unit, Radcliffe Infirmary, Oxford OX2 6HE, UK;* [3]*8612 Bunnell Drive, Potomac, MD 20854, USA; and* [4]*Department of Clinical Endocrinology, Imperial Cancer Research Fund, London WC2A 3PX, UK.*

**Summary** A case-control study was conducted to assess maternal hormonal factors associated with increased risk of bearing a cryptorchid son. Serum samples were collected during the first trimester of pregnancy from participants in the US Collaborative Perinatal Study. Twenty-five mothers of normal offspring (controls) were individually matched on medical center, age, parity, weight and length of gestation at the time of sampling to women bearing sons who had a diagnosis of cryptorchidism at one year of age or older. Compared with controls, mothers of cryptorchid sons (cases) had significantly greater percentages of non-protein bound ($P=0.010$) and albumin-bound ($P=0.014$) estradiol during the first trimester of the index pregnancy. On average, cases had 16% more bioavailable oestradiol than controls. Levels of human chorionic gonadotropin, testosterone, non-protein bound testosterone and sex-hormone binding globulin did not differ between the two groups. The data presented support the hypothesis that cryptorchidism results from elevated maternal oestrogen levels early in pregnancy.

Cryptorchidism, a relatively common abnormality of the male genitourinary system, is the major known risk factor for testicular cancer (Henderson *et al.*, 1979; Schottenfeld *et al.*, 1980; Depue *et al.*, 1983). Although the causes of both testicular cancer and cryptorchidism are largely unknown, their epidemiology (Depue *et al.*, 1983; Swerdlow *et al.*, 1983; Depue, 1984; Brown *et al.*, 1986) and the higher rates of urogenital developmental anomalies, including cryptorchidism, in testicular cancer cases and male family members of testicular cancer cases (Tollerud *et al.*, 1985) suggest common aetiological factors.

Higher maternal levels of oestrogen in early pregnancy may play a role in the aetiology of cryptorchidism. Testicular maldescent can be produced experimentally in animals by administering diethylstilbestrol (DES) or other forms of oestrogen during gestation (Jean, 1973; McLachlin *et al.*, 1975; Nomura & Kanzaki, 1977; Yasuda *et al.*, 1985). A number of clinical studies have found an increased frequency of cryptorchidism in males with a history of DES exposure *in utero* (Cosgrove *et al.*, 1977; Whitehead & Leiter, 1981). In epidemiologic studies of cryptorchidism, the abnormality has been associated with maternal use of exogenous oestrogens including DES during gestation (Gill *et al.*, 1979; Depue, 1984).

High maternal body weight also has been associated with an increased risk of cryptorchidism (Depue, 1984). Increased levels of 'free' (non-protein bound) oestradiol ($E_2$) are associated with high maternal body weight (Bernstein *et al.*, 1986). In pregnancy, plasma $E_2$ is mostly bound to sex-hormone binding globulin (SHBG) and the remainder to albumin, with only about 1% being free (Anderson, 1974). It is generally accepted that the non-protein bound $E_2$ is free to reach intracellular receptors, and there is now increasingly persuasive evidence that the $E_2$ bound to albumin may also be 'bioavailable' (Pardridge, 1986). The observed effect of high maternal body weight on risk of cryptorchidism may result from higher maternal levels of bioavailable $E_2$.

Another possible mechanism that has been suggested is that higher maternal $E_2$ levels exert an effect on testicular descent by lowering testosterone (T) levels in the foetus (Hadziselimovic & Herzog, 1980). If this is an important mechanism, high maternal T levels may protect against cryptorchidism.

Correspondence: L. Bernstein.
Received 15 April, 1988.

Davies *et al.* (1986) recently suggested that impaired placental function may play a role in cryptorchidism. They theorized that when levels of human chorionic gonadotropin (hCG) are reduced, there may be changes in foetal testicular function and hence an increased risk of maldescent.

The purpose of the present study was to determine whether first trimester maternal hormone levels in the index pregnancy differ between mothers of cryptorchid sons (cases) and mothers of 'normal' offspring (controls). Here, we compare levels of free and bound $E_2$, free and bound T, SHBG and hCG.

### Subjects and methods

### *Subjects*

Study subjects were participants in the Collaborative Perinatal Project of the National Institute of Neurological and Communicative Disorders and Stroke (Bethesda, MD, USA), a prospective study conducted to identify etiologic factors related to adverse pregnancy outcome (Niswander & Gordon, 1972). In this project, more than 55,000 pregnancies were registered at 12 university-affiliated medical centers in the United States between 1958 and 1965. The majority of these pregnancies were beyond 20 weeks (measured from day 1 of the last menstrual period) at the time of registration. Blood samples were collected at each prenatal visit. The samples have been stored at a central repository at $-20°C$. A detailed medical history was obtained from each participant and detailed obstetric and delivery records were kept.

In the Perinatal Project, offspring were examined at birth, 4 months, 1 year and 7 years of age; records were kept on congenital abnormalities and development. White women bearing sons who had a diagnosis of cryptorchidism at the 1 year or 7 year examination were considered eligible as 'cases' for the present study: no woman who had taken hormones or had experienced severe nausea or vomiting during the index pregnancy was considered eligible. There were 25 such women with sera available for evaluation who registered with the Perinatal Project by week 13 (measured from day 1 of the last menstrual period) of the relevant pregnancy. One control woman was individually matched to each case by medical center, race, parity, weight (within 4 kg), age at time of index pregnancy (within 5 years), and length of gestation at registration and sampling (within 11 days). Twenty-two of the 25 control mothers bore male offspring; for the remaining 3 cases, we were forced to use mothers of female offspring. All of the offspring of control mothers were followed for 7 years with no malformations noted.

*Assays*

Serum samples were shipped on dry ice to London (JM) for measurement of total $E_2$, percentage of $E_2$ bound to SHBG, percentage of free $E_2$, total T, percentage of free T, SHBG, and hCG. The identity of specimens was not known to the processing laboratory. The only identifier was a coded number unique for each submission of a specimen.

Ostradiol levels were measured by direct radioimmunoassay (Steranti Research Limited, St Albans, Herts., UK). The percentage of free $E_2$ was measured by centifugal-ultrafiltration-dialysis in undiluted serum at 37°C (Hammond et al., 1980). The percentage of $E_2$ which was bound to albumin was calculated from the measured free $E_2$ (%) in native serum and the free $E_2$ (%) observed in serum which had been heated at 60°C for 1 h (Hammond et al., 1982; Siiteri et al., 1982). SHBG was measured by a liquid phase immunoradiometric assay (Hammond et al., 1985) using antisera kindly supplied by Dr G.L. Hammond, University of Western Ontario, Canada. Levels of free T were measured by the 'Coat-A-Count' free testosterone kit method (Diagnostic Products Corporation, Los Angeles, CA, USA) and total T by direct assay using the Gamma-B $^{125}$I-testosterone kit (RIA UK, Washington, Tyne and Wear, UK). hCG was assayed by the double antibody kit method supplied by Diagnostic Products Corporation, Los Angeles, CA, USA.

*Statistical analysis*

The amount of free $E_2$ was computed as the product of total $E_2$ and the percentage of free $E_2$; other amounts were calculated in a similar manner. Hormone and SHBG values followed a lognormal distribution and logarithmic (base 10) values of these variables were used in all statistical analyses. Statistical analyses were performed using paired $t$ tests and repeated measures analysis of covariance. Adjustments for differences in length of gestation assumed a linear relationship between length of gestation and log hormone values. One-sided $P$ values are presented for these comparisons because the hormonal hypotheses to be tested predicted higher total (and bioavailable) $E_2$ which would be the stimulus for greater amounts of SHBG (Pearlman et al., 1967) and lower T and hCG levels.

### Results

One case-control pair was eliminated from the analyses that follow because the control's values for hCG and $E_2$ were low and not consistent with a 7 week gestation. Inclusion of this pair would have accentuated the differences presented below.

Relevant pregnancy characteristics and assay results for the remaining 24 matched case-control pairs are presented in Table I. Cases and controls were closely matched on age, weight and length of gestation at sampling. Subjects ranged in age from 18 to 39. Length of gestation at the time of sampling ranged from 46 to 93 days from the first day of the last menstrual period.

Although total $E_2$ concentrations of cases and controls did not differ, cases had significantly greater percentages of free $E_2$ ($P=0.010$) and of albumin-bound $E_2$ ($P=0.014$) than controls. The $E_2$ fractions that are considered biologically available were correspondingly greater in cases than in controls. On average, the cases had 16% more free $E_2$ ($P=0.066$) and 16% more albumin-bound $E_2$ ($P=0.038$) than controls. Levels of SHBG in cases and in controls were not significantly different. Total and free T levels did not differ significantly between cases and controls. Although not statistically significant, hCG levels were 15% higher in cases than in control women and this difference was consistently found across gestational ages.

Adjusting for length of gestation, parity, and, in the case of SHBG, for weight had no effect on the results presented in Table I. Results of analyses restricted to the 21 matched pairs in which all of the offspring were male did not alter the results presented.

**Table I** Relevant pregnancy characteristics (±s.d.) of study subjects and geometric mean hormone levels (log 10±s.d.) in early gestation for 24 mothers of cryptorchid sons (cases) and 24 mothers of normal offspring (controls)

| *Variable* | *Cases* | *Controls* | P-*value*[a] |
|---|---|---|---|
| Age (yr) | 25.7 (±5.4) | 25.0 (±5.0) | 0.080 |
| Weight (kg) | 60.2 (±17.1) | 59.1 (±13.0) | 0.497 |
| Days of gestation at sampling | 71.6 (±10.9) | 70.9 (±10.4) | 0.497 |
| Weeks of gestation at birth | 39.9 (±2.2) | 40.0 (±2.0) | 0.719 |
| Birth weight of offspring (g) | 3090 (±571) | 3313 (±632) | 0.146 |
| Oestradiol | | | |
| Total ($ng\,dl^{-1}$) | 969.2 (2.986±0.118) | 949.8 (2.978±0.167) | 0.413 |
| Binding percentages | | | |
| SHBG-bound[b] | 61.3 (±9.8) | 66.5 (±8.4) | 0.014 |
| Albumin-bound[b] | 37.6 (±9.6) | 32.5 (±8.3) | 0.014 |
| Free | 1.2 (±0.2) | 1.0 (±0.2) | 0.010 |
| Non-SHBG bound ($ng\,dl^{-1}$) | | | |
| Albumin-bound[b] | 348.1 (2.542±0.155) | 300.0 (2.477±0.163) | 0.038 |
| Free | 11.0 (1.042±0.157) | 9.5 (0.977±0.161) | 0.066 |
| Testosterone | | | |
| Total ($ng\,dl^{-1}$) | 125.9 (2.100±0.183) | 127.1 (2.104±0.204) | 0.471 |
| Free ($ng\,dl^{-1}$) | 3.4 (0.530±0.128) | 3.6 (0.561±0.130) | 0.219 |
| SHBG ($nmol\,l^{-1}$) | 212.3 (2.327±0.222) | 205.8 (2.313±0.216) | 0.392 |
| hCG ($IU\,ml^{-1}$) | 85.1 (1.930±0.278) | 74.2 (1.870±0.273) | 0.276 |

[a]Paired $t$-test, 2-sided $P$ values reported for pregnancy characteristics and 1-sided $P$ values reported for hormone and protein levels; [b]based on 22 pairs (insufficient samples available for 2 cases).

### Discussion

It is commonly accepted that testicular descent is under hormonal control (Hutson & Donahoe, 1986). We have previously hypothesized that excess endogenous maternal oestrogens play a role in the risk of cryptorchidism. In this study, we have found significantly greater percentages of free and albumin-bound $E_2$ in the first trimester sera of mothers bearing cryptorchid sons. This resulted in greater concentrations of non-SHBG bound $E_2$ in these pregnancies.

Burton *et al.* (1987) found no significant difference in maternal $E_2$ levels between mothers of cryptorchid boys and control mothers. They did not consider bioavailable $E_2$.

Two theories have been proposed to explain the effect of oestrogen on testicular descent. Hadziselimovic & Herzog (1980) proposed that the mechanism responsible for this effect is the suppression of foetal androgen secretion by oestrogen. It has, however, recently been shown in studies of hypogonadal mice that foetal testosterone production in early gestation is not relevant to the aetiology of cryptorchidism (Charlton, 1986; Grocock *et al.*, 1988). Current thinking (Hutson & Donahoe, 1986) considers a biphasic model for the hormonal control of testicular descent. In this model, separate hormones and mechanisms control the two stages of descent, the initial transabdominal phase, occurring prior to the twelfth week of gestation in man, and the transinguinal phase, occurring during the third trimester in man. The first stage is thought to be regulated by mullerian inhibiting substance, whereas the second, later stage is androgen dependent. Based on animal data, it appears that estrogens inhibit mullerian inhibiting substance (Newbold *et al.*, 1984; Hutson *et al.*, 1985) and cause atrophy of the gubernaculum (Wensing, 1973; Grocock *et al.*, 1988).

Although cryptorchidism is a risk factor for testis cancer, the risk of testis cancer is not confined to the involved testis in unilaterally cryptorchid men (Depue *et al.*, 1983). Cryptorchidism may be secondary to oestrogen inhibition of mullerian inhibiting substance resulting in intra-abdominal arrest of descent. Excess maternal oestrogen may mediate risk of testicular cancer more directly by interrupting the progression of primitive germ cells to mature germ cells. These primitive cells, persisting into puberty, would multiply under stimulation by gonadotropins and give rise to germ cell tumours of a variety of histological types depending on their particular stage of 'developmental arrest' (Henderson *et al.*, 1983).

This work was supported by grants CA 33512 and CA 00652 from the National Institutes of Health. The authors thank Dr John Sever for providing the sera and Dr Joseph Drage for providing the clinical data.

### References

ANDERSON, D.C. (1974). Sex-hormone-binding globulin. *Clin. Endocrinol.*, **3**, 69.

BERNSTEIN, L., DEPUE, R.H., ROSS, R.K., JUDD, H.L., PIKE, M.C. & HENDERSON, B.E. (1986). Higher maternal levels of free estradiol in first compared to second pregnancy: A study of early gestational differences. *J. Natl Cancer Inst.*, **76**, 1035.

BROWN, L.M., POTTERN, L.M. & HOOVER, R.N. (1986). Prenatal and perinatal risk factors for testicular cancer. *Cancer Res.*, **46**, 4812.

BURTON, M.H., DAVIES, T.W. & RAGGATT, P.R. (1987). Undescended testis and hormone levels in early pregnancy. *J. Epidemiol. Comm. Hlth.*, **41**, 127.

CHARLTON, H.M. (1986). Use of neural transplant to study neuroendocrine mechanisms. In *Frontiers in Neuroendocrinology*, Ganong, W.F. & Martini, L. (eds), vol. 9, p. 77. Raven Press: New York.

COSGROVE, M.D., BENTON, B. & HENDERSON, B.E. (1977). Male genitourinary abnormalities and maternal diethylstilbestrol. *J. Urol.*, **117**, 220.

DAVIES, T.W., WILLIAMS, D.R.R. & WHITAKER, R.H. (1986). Risk factors for undescended testis. *Int. J. Epidemiol.*, **15**, 197.

DEPUE, R.H. (1984). Maternal and gestational factors affecting the risk of cryptorchidism and inguinal hernia. *Int. J. Epidemiol.*, **13**, 311.

DEPUE, R.H., PIKE, M.C. & HENDERSON, B.E. (1983). Estrogen exposure during gestation and risk of testicular cancer. *J. Natl Cancer Inst.* **71**, 1151.

GILL, W.B., SCHUMACHER, G.F.B. & BIBBO, M. (1979). Pathological semen and anatomical abnormalities of the genital tract in human male subjects exposed to diethylstilbestrol *in utero*. *J. Urol.*, **117**, 477.

GROCOCK, C.A., CHARLTON, H.M. & PIKE, M.C. (1988). Role of the fetal pituitary in cryptorchidism induced by exogenous maternal oestrogen during pregnancy in mice. *J. Reprod. Fertil.*, **83**, 295.

HADZISELIMOVIC, F. & HERZOG, B. (1980). Etiology of testicular descent. *Clin. Androl.*, **3**, 166.

HAMMOND, G.L., LAHTEENMAKI, P.L.A., LAHTEENMAKI, P. & LUUKKAINEN, T. (1982). Distribution and percentages of non-protein bound contraceptive steroids in human serum. *J. Steroid Biochem.*, **17**, 375.

HAMMOND, G.L., LANGLEY, M.S. & ROBINSON, P.A. (1985). A liquid-phase immunoradiometric assay (IRMA) for human sex hormone binding globulin (SHBG). *J. Steroid Biochem.*, **23**, 451.

HAMMOND, G.L., NISKER, J.A., JONES, L.A. & SIITERI, P.K. (1980). Estimation of the percentage of free steroid in undiluted serum by centrifugal ultrafiltration-dialysis. *J. Biol. Chem.*, **255**, 5023.

HENDERSON, B.E., BENTON, B., JING, J., YU, M.C. & PIKE, M.C. (1979). Risk factors for cancer of the testis in young men. *Int. J. Cancer*, **23**, 598.

HENDERSON, B.E., ROSS, R.K., PIKE, M.C. & DEPUE, R.H. (1983). Epidemiology of testis cancer. In *Urological Cancer*, Skinner, D. (ed), p. 237. Grune: New York.

HUTSON, J.M. & DONAHOE, P.K. (1986). The hormonal control of testicular descent. *Endocrine Rev.*, **7**, 270.

HUTSON, J.M., DONAHOE, P.K. & MACLAUGHLIN, D.T. (1985). Steroid modulation of mullerian duct regression in the chick embryo. *Gen. Comp. Endocrinol.*, **57**, 88.

JEAN, C. (1973). Croissance et structure des testicules cryptorchides chez les souris nees de meres truitees a l'obstetractiol parchant la gestation. *Ann. Endocrinol. (Paris)*, **34**, 669.

McLACHLAN, J.A., NEWBOLD, R.R. & BULLOCK, B. (1975). Reproductive tract lesions in male mice exposed prenatally to diethylstilbestrol. *Science*, **190**, 991.

NEWBOLD, R.R., SUZUKI, Y. & McLACHLAN, J.A. (1984). Mullerian duct maintenance in heterotypic organ culture after *in vivo* exposure to diethylstilbestrol. *Endocrinology*, **115**, 1863.

NISWANDER, N.R. & GORDON, M. (1972). *The Women and Their Pregnancies*. W.B. Saunders: Philadelphia.

NOMURA, T. & KANZAKI, T. (1977). Induction of urogenital anomalies and some tumors in the progeny of mice receiving diethylstilbestrol during pregnancy. *Cancer Res.*, **37**, 1099.

PARDRIDGE, W.M. (1986). Serum bioavailability of sex-steroid hormones. *Clinics Endocrinol. Metabol.*, **15**, 259.

PEARLMAN, W.H., CREPY, O. & MURPHY, M. (1967). Testosterone-binding levels in the serum of women during the normal menstrual cycle, pregnancy and the post-partum period. *J. Clin. Endocrin. Metab.*, **27**, 1012.

SCHOTTENFELD, D., WARSHAUER, M.E., SHERLOCK, S., ZAUBER, A.G., LEDER, M. & PAYNE, R. (1980). The epidemiology of testicular cancer in young adults. *Am. J. Epidemiol.*, **112**, 232.

SIITERI, P.K., MURAI, J.T., HAMMOND, G.L., NISKER, J.A., RAYMOURE, W.J. & KUHN, R.W. (1982). The serum transport of steroid hormones. *Recent Prog. Horm. Res.*, **38**, 457.

SWERDLOW, A.J., WOOD, K.H. & SMITH, P.G. (1983). A case-control study of the aetiology of cryptorchidism. *J. Epidemiol. Commun. Health*, **37**, 238.

TOLLERUD, D.J., BLATTNER, W.A., FRASER, M.C. & 9 others (1985). Familial testicular cancer and urogenital developmental anomalies. *Cancer*, **55**, 1849.

WENSING, C.J.G. (1973). Testicular descent in some domestic animals. III: Search for the factors that regulate the gubernacular reaction. *Proc. Kon. Ned. Akad. Wetensch.*, **76**, 196.

WHITEHEAD, D.E. & LEITER, E. (1981). Genital abnormalities and abnormal semen analyses in male patients exposed to diethylstilbestrol *in utero*. *J. Urol.*, **125**, 47.

YASUDA, Y., KIHARA, T., TANIMURA, T. & NISHIMURA, H. (1985). Gonadal dysgenesis induced by prenatal exposure to ethinyl estradiol in mice. *Teratology*, **21**, 219.